# Effect of red and blue light versus white light on fruit biomass radiation-use efficiency in dwarf tomatoes

**DOI:** 10.3389/fpls.2024.1393918

**Published:** 2024-06-21

**Authors:** Xinglin Ke, Hideo Yoshida, Shoko Hikosaka, Eiji Goto

**Affiliations:** ^1^ Graduate School of Horticulture, Chiba University, Matsudo, Chiba, Japan; ^2^ Research Center for Space Agriculture and Horticulture, Chiba University, Chiba, Matsudo, Japan

**Keywords:** blue light, indoor farming, micro-tom, plant factory, red light, vertical farming

## Abstract

The effect of the ratio of red and blue light on fruit biomass radiation-use efficiency (FBRUE) in dwarf tomatoes has not been well studied. Additionally, whether white light offers a greater advantage in improving radiation-use efficiency (RUE) and FBRUE over red and blue light under LED light remains unknown. In this study, two dwarf tomato cultivars (‘Micro-Tom’ and ‘Rejina’) were cultivated in three red-blue light treatments (monochromatic red light, red/blue light ratio = 9, and red/blue light ratio = 3) and a white light treatment at the same photosynthetic photon flux density of 300 μmol m^–2^ s^–1^. The results evidently demonstrated that the red and blue light had an effect on FBRUE by affecting RUE rather than the fraction of dry mass partitioned into fruits (F_fruits_). The monochromatic red light increased specific leaf area, reflectance, and transmittance of leaves but decreased the absorptance and photosynthetic rate, ultimately resulting in the lowest RUE, which induced the lowest FBRUE among all treatments. A higher proportion of blue light (up to 25%) led to a higher photosynthetic rate, resulting in a higher RUE and FBRUE in the three red-blue light treatments. Compared with red and blue light, white light increased RUE by 0.09–0.38 g mol^−1^ and FBRUE by 0.14–0.25 g mol^−1^. Moreover, white light improved the F_fruits_ in ‘Rejina’ and Brix of fruits in ‘Micro-Tom’ and both effects were cultivar-specific. In conclusion, white light may have greater potential than mixed red and blue light for enhancing the dwarf tomato FBRUE during their reproductive growth stage.

## 1 Introduction

Cultivation of dwarf tomatoes in a plant factory with artificial light (PFAL), also known as a vertical farm, offers numerous advantages (Kato et al., 2011; Ke et al., 2021). In comparison with general tomato cultivars, the plant density (Meissner et al., 1997) and space-use efficiency of the dwarf tomato are higher, and its growth cycle is shorter (Sun et al., 2006). However, half of the energy cost in a PFAL is used for lighting (Ohyama et al., 2002; Graamans et al., 2018). Hence, cultivating dwarf tomatoes sustainably in a PFAL to improve radiation-use efficiency (RUE) is crucial. Additionally, particular attention should be given to improving fruit biomass radiation-use efficiency (FBRUE) in commercial PFALs producing tomato fruits. FBRUE is defined as the ratio of the dry mass of a plant’s fruits to the number of photosynthetic photons captured by the plant (Wheeler et al., 2008; Li et al., 2019; Ke et al., 2023) and calculated as the product of RUE and the fraction of dry mass partitioned into fruits (Ke et al., 2023). In a PFAL, light quality can be manipulated and regulated to not only improve biomass yield and quality (Goto, 2003; Ilić and Fallik, 2017; Ji et al., 2020) but also potentially upgrade FBRUE by enhancing RUE and/or the fraction of dry mass partitioned into fruits in tomatoes.

According to McCree (1972a), many plants exhibit the highest quantum efficiency of absorption in the red and blue wavelength ranges, making red and blue light highly efficient in PFALs. Red and blue light can also affect the morphology and photosynthesis of tomatoes. Red light can increase plant height (Liu et al., 2011; Nanya et al., 2012), increase leaf area (Bugbee, 2016), and reduce specific leaf area (SLA), while blue light can reduce plant height and increase SLA (Snowden et al., 2016; Ke et al., 2021; Kong and Nemali, 2021). However, the effect of blue light on photosynthesis varies with cultivars in tomatoes (Ouzounis et al., 2016). The ratio of red and blue light may affect the RUE of the canopy by affecting the photosynthetic quantum yield of the canopy and leaf (Ke et al., 2021), as well as the dry matter distribution, such as increasing the shoot/root ratio (Goto, 2003; Nanya et al., 2012). Therefore, this ratio may have an effect on FBRUE by affecting RUE and the fraction of dry mass partitioned into fruits. However, to date, no study has investigated the effect of the ratio of red and blue light on the FBRUE of dwarf tomatoes.

Recently, white LEDs are increasingly being utilized in PFALs owing to their advantages, which include low price, wide spectrum range, and creating a more comfortable working environment. More than 60% of horticultural lighting devices use white LEDs (Kusuma et al., 2020). Moreover, white light not only contains red and blue light but also green light and far-red light. The transmittance of green light is high, allowing leaves in the lower canopy to absorb light, which enhances the uniformity of light distribution in the canopy. Additionally, far-red light can increase the photosynthetic rate (Pn) (Zhen and van Iersel, 2017; Kalaitzoglou et al., 2019), and a previous study demonstrated that it could increase the allocation of dry matter to fruits (Ji et al., 2019); therefore, compared with red and blue light, white light may increase FBRUE. However, to the best of our knowledge, there is not much information on the comparison of FBRUE in dwarf tomatoes grown under white light and red-blue light.

This study aimed to investigate the effect of the ratio of red and blue light on FBRUE in dwarf tomatoes. Additionally, we aimed to verify whether FBRUE under white light is higher than that under red and blue light and to determine an appropriate light quality to improve FBRUE at the reproductive growth stage. In this study, we quantified the effects of light quality on FBRUE, RUE, and dry matter partitioning of fruits by using white light and blue and red light with three different red/blue light ratios.

## 2 Materials and methods

### 2.1 Plant materials and growth conditions

Two dwarf tomato cultivars, ‘Micro-Tom’ and ‘Rejina’ (*Lycopersicon esculentum*), were used as the test materials. After 3-day germination, at a photosynthetic photon flux density (PPFD) of 200 μmol m^–2^ s^–1^, we cultivated tomato seeds using white light (LDL40S-N19/21, Panasonic Corporation, Osaka, Japan) in a cultivation room with the following environmental conditions: 1000 μmol mol^–1^ CO_2_ concentration, 25/20°C (day/night) air temperature, 70% relative humidity, and 16/8 h (day/night) photoperiod.

At 24 days after sowing (DAS), all seedlings were transplanted under red and blue LED lamps (CIVILIGHT, DPT2RB120Q33 40 type, Showa Denko K.K., Tokyo, Japan; red:blue = 9:1), and PPFD above the top of the canopy was set as 300 μmol m^–2^ s^–1^. As the growth rate and anthesis time of the first flower in the two cultivars were distinct, the plant density management between the two cultivars was different. The number of days, plant density, used lamps, and PPFD on the top of the canopy during growth periods are shown in **Supplementary Table 1**. The first flowers of half of the plants in ‘Micro-Tom’ and ‘Rejina’ bloomed at 36 and 50 DAS, respectively. Finally, when the experiments started, the values of leaf area (LA) / projected leaf area (PLA) in ‘Micro-Tom’ at 36 DAS and in ‘Rejina’ at 50 DAS were modulated at 1.5 and 1.6, respectively (**Supplementary Figure 1**).

Following 36 DAS in ‘Micro-Tom’ and 50 DAS in ‘Rejina’, the plants were placed in four treatments with different light qualities (**Supplementary Figure 2**): red light (R), white light (WH, the same lamps as previously described white LED), and the mixture of red and blue lights: red/blue light ratio = 3 (R3B1) and red/blue light ratio = 9 (R9B1). The PPFD above the top of all canopies was set at 300 μmol m^–2^ s^–1^. Apart from the light condition, other environmental conditions remained unchanged. As soon as visible side shoots and axillary buds appeared, all plants were pruned. Final harvests were conducted when half of the fruits turned red at 82 DAS in ‘Micro-Tom’ and at 100 DAS in ‘Rejina’, respectively. The spectral photon flux distributions of the LED lamps are shown in **Supplementary Figure 3** and calculations were performed for the blue, green, and red wavelength fractions (**Supplementary Table 2**).

### 2.2 Growth measurement, Brix, and acidity of fruits

In each treatment, three to four plants were sampled for fresh and dry biomass analysis at 36, 43, 50, 57, 64, 71, and 82 DAS for ‘Micro-Tom’ and at 50, 60, 70, 80, 90, and 100 DAS for ‘Rejina’. A leaf area meter (LI-3000C, Li-Cor Inc., Lincoln, NE, USA) was utilized to measure LA in ‘Micro-Tom’ and ‘Rejina’ at 82 and 100 DAS, respectively, with the results taken from two replicates. Specific leaf area (SLA, cm^2^ g^−1^) was calculated by dividing the LA (cm^2^) by the leaf dry weight (g). Additionally, the number of fruits was recorded and plant height was measured.

At 82 DAS in ‘Micro-Tom’ and 100 DAS in ‘Rejina’, two parameters of fruit quality (Brix and acidity level) were determined using a pocket Brix-Acidity Meter (PAL-BX|ACID3; Atago Co. Ltd.) in 6−9 ripe tomatoes from three or four plants for each treatment.

### 2.3 Reflectance, transmittance, absorptance, Pn and chlorophyll concentration of leaves

The reflection and transmission spectra of leaves at 50, 71, and 82 DAS for ‘Micro-Tom’ and 50, 60, 70, and 80 DAS for ‘Rejina’ were measured with three plants sampled per treatment using the same method reported by Ke et al. (2023). The absorptance was then computed by subtracting the reflectance and transmittance from 100%.

At 53, 67, and 81 DAS, the Pn of the topmost, fully expanded, and unshaded leaf of three randomly selected plants in each treatment was determined using a portable photosynthesis measurement system (LI-6400XT, LI-COR Inc., Lincoln, NE, USA) under the same environmental conditions shown in the article (Ke et al., 2023).

The chlorophyll concentration was determined on a dry weight basis using an ultraviolet-visible spectrophotometer (V-750, JASCO Corporation, Tokyo, Japan) and was extracted from the first leaf from the top of the main stem with N,N-dimethylformamide at 50, 71 and 82 DAS in ‘Micro-Tom’ and at 50, 70, 80, and 100 DAS in ‘Rejina’, according to the protocol and method of Porra et al. (1989). Three or four plants (one leaf per plant) in each treatment were sampled.

### 2.4 Radiation-use efficiency (RUE) and fruit biomass radiation-use efficiency (FBRUE)

RUE (g mol^−1^) is the proportion of the accumulated dry mass (ΔW, g) to the integrated PPFD received by a plant during a given period (ΔI_PPFD_, mol) using projected leaf area (Ke et al., 2023). In this study, the RUE remained constant through the reproductive growth stage. Thus, the gradient of the fitted linear regression expressing the connection between ΔW and ΔI_PPFD_ was the value of RUE. FBRUE is defined as RUE (g mol^–1^) multiplied by the fraction of dry mass partitioned into fruits (F_fruits_, g g^–1^) on a given day (Ke et al., 2023).

### 2.5 Statistical analysis

Data analysis was performed using SPSS for Windows (Version 24.0; SPSS Inc., Chicago, IL, USA). The Tukey–Kramer test at *p* < 0.05 was conducted to investigate significant differences among treatments after performing one-way analysis of variance (ANOVA) on the data. The mean values of measured data were compared, and the measurements related to FBRUE in each treatment were repeated three times.

## 3 Results

### 3.1 Growth condition

Light quality significantly affected the plant height of ‘Rejina’ but not ‘Micro-Tom’ (**Table 1**). The plant height in R was significantly higher than that in WH. Additionally, light quality affected the SLA in the two cultivars. The SLA of the two cultivars in R without blue light was the highest and significantly higher than those in R3B1, which had the highest blue light ratio. Light quality also had significant effects on the total dry weight ratio in ‘Micro-Tom’. However, total fresh and dry weights in the two cultivars were not significantly affected by light quality.

**Table 1:** Effect of light quality on the growth in ‘Micro-Tom’ 82 days after sowing (DAS) and in ‘Rejina’ 100 DAS.

Cultivar	Initial value or treatment	Plant height (cm)	Specific leaf area (cm^2^ g^−1^ DW)	Total fresh weight (g)	Total dry weight (g)	Total dry matter ratio (%)
Micro-Tom	Initial value at 36 DAS	10.3 ± 0.4	288.5 ± 13.7	10.2 ± 0.7	0.9 ± 0.1	8.9 ± 0.3
	R	12.7 ± 0.7	157.2 ± 17.4 a	118.8 ± 12.7	10.4 ± 1.6	8.6 ± 0.5 b
	R9B1	12.1 ± 0.3	133.0 ± 8.5 ab	115.0 ± 9.8	10.9 ± 0.9	9.5 ± 0.4 ab
	WH	11.3 ± 0.3	115.7 ± 4.8 ab	104.6 ± 17.4	10.9 ± 1.8	10.5 ± 0.1 a
	R3B1	13.2 ± 0.2	109.0 ± 1.6 b	133.9 ± 5.8	13.1 ± 0.5	9.8 ± 0.2 ab
Rejina	Initial value at 50 DAS	13.7 ± 0.4	186.9 ± 11.9	69.4 ± 2.0	6.5 ± 0.2	9.3 ± 0.2
	R	21.7 ± 1.7 a	136.4 ± 6.8 a	382.2 ± 67.0	31.2 ± 4.5	8.3 ± 0.6
	R9B1	18.5 ± 0.8 ab	113.6 ± 1.3 b	416.8 ± 85.3	34.7 ± 5.7	8.5 ± 0.3
	WH	16.5 ± 0.3 b	102.0 ± 3.4 b	435.7 ± 57.7	35.4 ± 5.1	8.1 ± 0.1
	R3B1	19.0 ± 0.8 ab	110.3 ± 4.2 b	452.4 ± 91.1	37.0 ± 6.0	8.4 ± 0.4

DW is dry weight (g). Each value represents the mean ± standard error. Different letters in a column in a cultivar indicate significant differences among the treatments based on Tukey−Kramer’s test at *p* < 0.05 (n = 6−8). R, red light; R9B1, red/blue light ratio = 9; WH, white light; R3B1, red/blue light ratio = 3.

### 3.2 Reflectance, transmittance, and absorptance of leaves

Photosynthetically active radiation was most reflected and least absorbed by the top leaves in R in the two cultivars (**Tables 2**, **3**). In ‘Micro-Tom’, the reflectance values to green and red light in R were significantly higher than those in WH (**Table 2**). Moreover, the absorptance values to green and red light in R were significantly lower than those in other treatments. In ‘Rejina’, the transmittance of leaves in R was significantly higher than those in other treatments (**Table 3**).

**Table 2:** Effects of light quality on the reflectance and absorptance of leaves in ‘Micro-Tom’ at the green and red wavelengths 82 DAS.

Treatment	Reflectance (%)		Absorptance (%)	
	500–599 nm (Green)	600–700 nm (Red)	500–599 nm (Green)	600–700 nm (Red)
R	9.3 ± 0.0 a	7.2 ± 0.0 a	86.1 ± 1.2 b	90.9 ± 0.6 b
R9B1	6.3 ± 0.0 ab	5.0 ± 0.1 ab	90.8 ± 0.8 a	94.0 ± 0.3 a
WH	5.2 ± 1.4 b	3.8 ± 1.4 b	89.4 ± 0.4 a	94.0 ± 0.9 a
R3B1	6.1 ± 0.4 ab	4.8 ± 0.0 ab	91.8 ± 0.7 a	93.1 ± 0.0 a

The range of measured light spectrum was 400–700 nm. Each value represents the mean ± standard error. Different letters in a column in a cultivar indicate significant differences among the treatments based on Tukey−Kramer’s test at *p* < 0.05 (n = 3−4). R, red light; R9B1, red/blue light ratio = 9; WH, white light; R3B1, red/blue light ratio = 3.

**Table 3:** Effects of light quality on the reflectance, transmittance and absorptance of leaves in ‘Rejina’ at the blue, green and red wavelengths 100 DAS.

Treatment	Reflectance (%)			Transmittance (%)			Absorptance (%)		
	400–499 nm (Blue)	500–599 nm (Green)	600–700 nm (Red)	400–499 nm (Blue)	500–599 nm (Green)	600–700 nm (Red)	400–499 nm (Blue)	500–599 nm (Green)	600–700 nm (Red)
R	5.6 ± 0.1	8.9 ± 0.4 a	7.7 ± 0.5 a	0.5 ± 0.1 a	5.7 ± 0.7 a	3.3 ± 0.4 a	94.1 ± 0.1 b	85.7 ± 0.6 b	88.7 ± 0.2 b
R9B1	5.5 ± 0.0	6.5 ± 0.1 b	6.2 ± 0.1 ab	0.1 ± 0.0 b	1.8 ± 0.4 b	0.9 ± 0.2 b	94.5 ± 0.0 ab	91.7 ± 0.5 a	92.9 ± 0.3 a
WH	5.0 ± 0.3	5.9 ± 0.1 b	5.3 ± 0.5 b	0.1 ± 0.0 b	2.5 ± 0.6 b	1.3 ± 0.2 b	94.9 ± 0.3 ab	91.6 ± 0.6 a	93.4 ± 0.5 a
R3B1	4.7 ± 0.3	5.8 ± 0.5 b	5.1 ± 0.0 b	0.1 ± 0.0 b	1.7 ± 0.1 b	1.6 ± 0.0 b	95.3 ± 0.3 a	92.5 ± 0.4 a	93.3 ± 0.0 a

The range of measured light spectrum was 400–700 nm. Each value represents the mean ± standard error. Different letters in a column in a cultivar indicate significant differences among the treatments based on Tukey−Kramer’s test at *p* < 0.05 (n = 3−4). R, red light; R9B1, red/blue light ratio = 9; WH, white light; R3B1, red/blue light ratio = 3.

### 3.3 Pn and chlorophyll concentration

**Figure 1:**
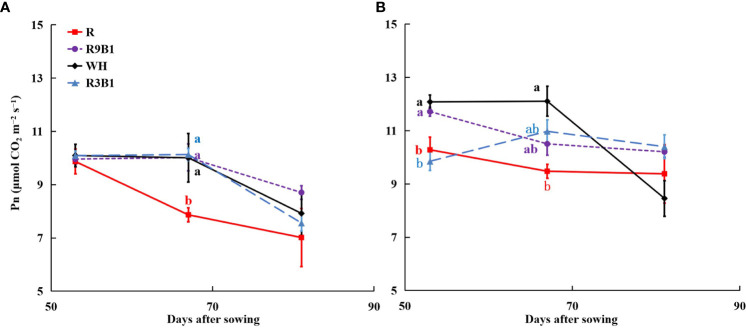
Effects of light quality on net photosynthetic rate (Pn) in ‘Micro-Tom’ **(A)** and ‘Rejina’ **(B)** at 53, 67, and 81 DAS. Solid points represent the average Pn of three or four plants in each treatment. Error bars represent ± standard error. Different letters indicate significant differences among the treatments based on Tukey−Kramer’s test at *p* < 0.05 (n = 3−4). R, red light; R9B1, red/blue light ratio = 9; WH, white light; R3B1, red/blue light ratio = 3; DAS, days after sowing.

The values of Pn in R at 53, 67, and 82 DAS were the lowest among all treatments in ‘Micro-Tom’ (**Figure 1A**). At 67 DAS, the Pn in R was significantly lower than that in other treatments. Similar to ‘Micro-Tom’, the values of Pn in ‘Rejina’ under red light were significantly lower than those in WH at 53 and 67 DAS (**Figure 1B**).

**Figure 2:**
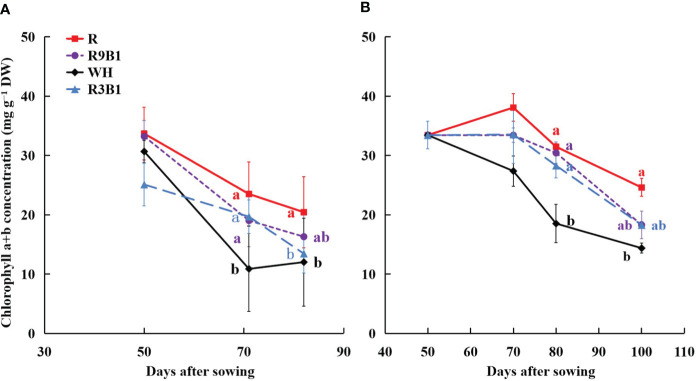
Effects of light quality on chlorophyll concentration of leaves in ‘Micro-Tom’ **(A)** at 50, 71, and 82 DAS, and in ‘Rejina’ **(B)** at 50, 70, 80, and 100 DAS. DW **(g)** is dry weight. Solid points represent the average value of three plants in each treatment. Error bars represent ± standard error. Different letters indicate significant differences among the treatments based on Tukey−Kramer’s test at *p* < 0.05 (n = 3). R, red light; R9B1, red/blue light ratio = 9; WH, white light; R3B1, red/blue light ratio = 3; DAS, days after sowing.

Light quality significantly affected the concentration of chlorophyll a+b in the two cultivars (**Figure 2**). The chlorophyll concentration of leaves in ‘Micro-Tom’ in WH was significantly lower than that in other treatments at 71 DAS (**Figure 2A**). At 82 DAS, it was significantly higher in R than in WH and R3B1. Similar to ‘Micro-Tom’, the chlorophyll concentration of leaves in ‘Rejina’ in WH at 80 DAS was significantly lower than that in other treatments (**Figure 2B**). Additionally, at 100 DAS, the chlorophyll concentration of leaves in R was significantly higher than WH.

### 3.4 RUE and FBRUE

**Figure 3:**
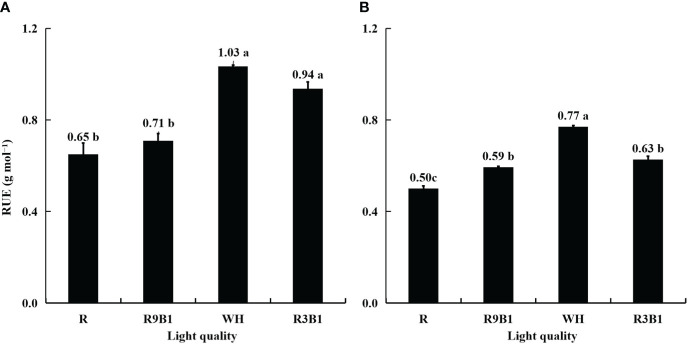
Effects of light quality on RUE in ‘Micro-Tom’ **(A)** and ‘Rejina’ **(B)** during the reproductive growth stage. The RUE was calculated using the data in **Supplementary Figures 4**, **5**. Error bars represent ± standard error. Different letters indicate significant differences among the treatments based on Tukey−Kramer’s test at *p* < 0.05 (n = 3). R, red light; R9B1, red/blue light ratio = 9; WH, white light; R3B1, red/blue light ratio = 3.

The RUE in **Figure 3** was calculated using the data in **Supplementary Figures 4**, **5**. In ‘Micro-Tom’, the values of RUE in WH and R3B1 were significantly higher than those in R and R9B1 (**Figure 3A**). The highest RUE in WH was 0.38 g mol^−1^ higher than the lowest RUE in R. Similarly, in ‘Rejina’, the RUE value in WH was the highest, and that in R was the lowest among all treatments (**Figure 3B**). The blue light proportions of R, R9B1, and R3B1 were 0%, 10%, and 25%, respectively (**Supplementary Table 2**). The RUE increased with an increase in the blue light proportion from 0% to 25% under red and blue light in both cultivars (**Figure 3**).

**Figure 4:**
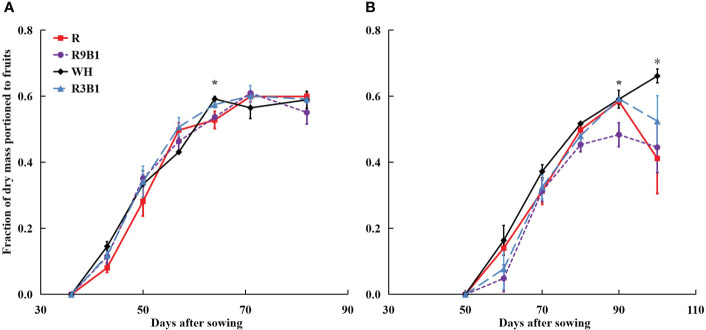
Effects of light quality on the fraction of dry mass portioned to fruits (F_fruits_) over time in ‘Micro-Tom’ **(A)** and ‘Rejina’ **(B)**. Error bars represent ± standard error. * indicates significant differences among the treatments based on Tukey−Kramer’s test at *p* < 0.05 (n = 3−6). R, red light; R9B1, red/blue light ratio = 9; WH, white light; R3B1, red/blue light ratio = 3.

In ‘Micro-Tom’, F_fruits_ increased from 36 to 71 DAS in all treatments and did not change until 82 DAS (**Figure 4A**). At 64 DAS, the F_fruits_ values in WH and R3B1 were significantly higher than F_fruits_ in R and R9B1, temporarily. Finally, no significant difference was observed in F_fruits_ at 82 DAS (harvest time) among treatments. In ‘Rejina’, F_fruits_ increased from 50 to 90 DAS in all treatments and decreased until 100 DAS except in WH (**Figure 4B**). The F_fruits_ values in WH and R3B1 were significantly higher than those in R9B1 at 90 DAS. Moreover, the F_fruits_ in WH were significantly higher than those in other treatments at 100 DAS.

**Figure 5:**
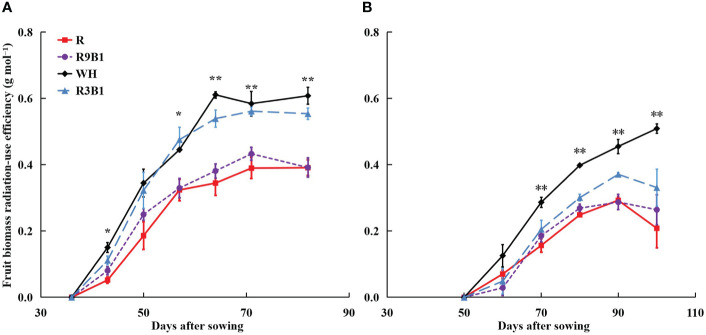
Effects of light quality on fruit biomass radiation-use efficiency (FBRUE) over time in ‘Micro-Tom’ **(A)** and ‘Rejina’ **(B)**. Error bars represent ± standard error. * and ** indicates significant difference among the treatments based on Tukey−Kramer’s test at *p* < 0.05 and *p* < 0.01 (n = 3), respectively. R, red light; R9B1, red/blue light ratio = 9; WH, white light; R3B1, red/blue light ratio = 3.

In ‘Micro-Tom’, FBRUE was significantly affected by light quality at 43, 57, 64, 71, and 82 DAS (**Figure 5A**). The FBRUE values in WH and R3B1 were significantly higher than those in R and R9B1 from 64 DAS. In ‘Rejina’, FBRUE was affected by light quality from 70 DAS; specifically, the FBRUE in WH was significantly greater than that observed in other treatments (**Figure 5B**).

### 3.5 Yield, Brix and acidity of fruits

In ‘Micro-Tom’, there were no significant differences in fruit fresh and dry weights among the treatments at 71 and 82 DAS (**Supplementary Tables 3**, **4**). At 82 DAS, the fruit dry matter ratio in WH was significantly higher than that in R. Additionally, Brix in WH was significantly higher than that in R3B1 (**Table 4**). In ‘Rejina’, at 70 DAS, fruit fresh and dry weights in WH were the highest among all treatments and significantly higher than those in R (**Supplementary Table 3**). However, at 100 DAS, no significant differences were observed in all items among treatments (**Table 4**; **Supplementary Table 4**).

**Table 4:** Effects of light quality on the fruit dry matter ratio and fruit quality in ‘Micro-Tom’ 82 DAS and in ‘Rejina’ 100 DAS.

Cultivar	Treatment	Fruit dry matter ratio (%)	Brix (%)	Acidity (%)	Brix/acidity
Micro-Tom	R	7.7 ± 0.4 b	5.4 ± 0.1 ab	1.2 ± 0.1	4.7 ± 0.3
	R9B1	8.3 ± 0.4 ab	5.3 ± 0.2 ab	1.2 ± 0.1	4.5 ± 0.4
	WH	9.6 ± 0.1 a	5.7 ± 0.4 a	1.3 ± 0.1	4.5 ± 0.3
	R3B1	8.5 ± 0.2 ab	4.8 ± 0.2 b	1.3 ± 0.1	4.1 ± 0.6
Rejina	R	6.5 ± 0.4	5.5 ± 0.1	0.7 ± 0.1	8.5 ± 0.8
	R9B1	6.5 ± 0.1	5.5 ± 0.2	0.7 ± 0.1	7.9 ± 0.6
	WH	5.8 ± 0.2	5.9 ± 0.2	0.6 ± 0.0	10.6 ± 0.8
	R3B1	6.8 ± 0.1	5.8 ± 0.1	0.7 ± 0.0	8.1 ± 0.5

Each value represents the mean ± standard error. Different letters indicate significant differences at the *p* < 0.05 level among light-quality treatments with Tukey−Kramer’s test. Each value of the fruit dry matter ratio represents a mean of three values. There were 6−9 fruits sampled in each treatment for fruit quality. R, red light; R9B1, red/blue light ratio = 9; WH, white light; R3B1, red/blue light ratio = 3.

## 4 Discussion

### 4.1 Influence of the proportion of red and blue light on RUE due to alterations in leaf optical characteristics and photosynthesis

In the present study, monochromatic red light increased SLA (**Table 1**) and chlorophyll concentration (**Figure 2**) compared with combined red-blue light and white light. In corn, there is an inverse correlation between chlorophyll concentrations and the reflectance of green and red light (Daughtry et al., 2000). Moreover, higher SLA leads to thinner leaves with the same dry matter ratio and higher transmittance of leaves. Consequently, the monochromatic red light increased the reflectance and transmittance but decreased the absorptance (**Tables 2**, **3**) and may ultimately cause the over-valuation of the ΔI_PPFD_ and the under-valuation of RUE. Additionally, there were no significant differences in leaf optical properties among all treatments at 50 and 71 DAS in ‘Micro-Tom’ and 50, 70, and 80 DAS in ‘Rejina’ (data not shown). Therefore, the effects of light quality on leaf optical properties appeared significantly in the late period of the reproductive growth stage.

RUE is affected by the ratio of red and blue light not only because of its effect on optical properties but also its effect on photosynthesis. For optimal plant growth, the addition of at least a low percentage of blue light to supplement red light is necessary (Hoenecke et al., 1992; Cope and Bugbee, 2013). Monochromatic red light decreased the RUE (**Figure 3**) by decreasing the Pn (**Figure 1**). Several crop plants have demonstrated a decreased photosynthesis rate when grown solely under red light, such as rice (Matsuda et al., 2004), wheat (Goins et al., 1997), cucumber (Hogewoning et al., 2010), and radish (Yorio et al., 2001). This may be because the disruption to the photosynthetic machinery is caused by the presence of red light only or the lack of blue light (Hogewoning et al., 2010). Additionally, monochromatic red light results in low F_v_/F_m_ in cucumber (Hogewoning et al., 2010) and the inhibition of PSI and PSII development in wheat (Sood et al., 2004).

Under the combined red and blue light, a higher blue light proportion, up to 25%, resulted in a higher Pn at 67 DAS in ‘Rejina’ (**Figure 1B**). This may be associated with the decreasing SLA (**Table 1**) and stomatal conductance and an increase in photosynthetic electron transport capacity (Miao et al., 2016; Izzo et al., 2020). However, there were no significant differences in total dry weights between the two cultivars (**Table 1**). This result contrasts with the finding that a large proportion of blue light has the potential to hinder the production of biomass in tomato seedlings (cv. Early girl) at PPFDs of 200 and 500 μmol m^–2^ s^–1^ (Snowden et al., 2016). However, in the same study, there were no significant differences in dry mass among different light qualities in cucumber at a PPFD of 200 μmol m^–2^ s^–1^ as well as in radish, soybean, lettuce (cv. Waldmann’s Green), and wheat at PPFDs of 200 and 500 μmol m^–2^ s^–1^. The dry mass of lettuce plants “Gentilina” (cv. Rebelina) decreased and then increased with an improved proportion of blue light at a PPFD of 215 μmol m^–2^ s^–1^ (Pennisi et al., 2019). This may be attributed to the highly cultivar-specific effects of the ratio of red and blue light on stomatal conductance and Pn in tomatoes (Ouzounis et al., 2016). Therefore, at 67 DAS, there was a significant difference in Pn between monochromatic red light and mixed red and blue light in ‘Micro-Tom’ (**Figure 1A**) but no significant difference in ‘Rejina’ (**Figure 1B**). Additionally, the values of Pn in the two cultivars decreased with time. This may be attributed to leaf senescence (Quirino et al., 2000). Thus, a higher blue light proportion led to higher Pn and further led to higher RUE (**Figure 3**) under the combination of red and blue light.

### 4.2 Blue light improves FBRUE by improving RUE rather than F_fruits_

Except for two temporary periods around 64 DAS in ‘Micro-Tom’ and 90 DAS in ‘Rejina’, there was no significant difference in F_fruits_ among the three treatments under the combination of red and blue light (**Figure 4**). Until 71 DAS in ‘Micro-Tom’ and 70 DAS in ‘Rejina’, a higher blue light proportion resulted in higher fruit fresh and dry weights among the three treatments (**Supplementary Table 3**). However, both cultivars are determinate tomatoes, and no new fruits emerged from the main stem during the late reproductive growth stage. Hence, there were no significant differences in the number of fruits and fruit fresh and dry weights among the three treatments until the harvest (**Supplementary Table 4**). Consequently, blue light improved FBRUE by mainly increasing RUE rather than F_fruits_.

The range of FBRUE in ‘Micro-Tom’ was 0.39–0.61 g mol^−1^, which was higher than 0.21–0.33 g mol^−1^ in ‘Rejina’ (**Figure 5**). Previous studies have demonstrated that the FBRUE values of tomatoes cultivated in a controlled environment ranged from 0.20−0.36 g mol^−1^ (Goto, 2011; Li et al., 2019), which was almost the same as for ‘Rejina’ in the present study. The F_fruits_ values in ‘Micro-Tom’ and ‘Rejina’ were 0.55−0.60 and 0.41−0.66 g mol^−1^, respectively (**Figure 4**) which were similar to the values reported perviously (Cockshull et al., 1992; De Koning, 1993; Cavero et al., 1998; Scholberg et al., 2000). The range of RUE in ‘Micro-Tom’ was 0.65–1.03 g mol^−1^, which was higher than the RUE of 0.50–0.77 g mol^−1^ in ‘Rejina’ (**Figure 3**). Therefore, the difference in FBRUE between the two cultivars was mainly due to the distinction in RUE rather than F_fruits_.

Although the two cultivars could not be compared statistically because of the inconsistency in plant density and light environment, the RUE of ‘Micro-Tom’ was higher than that of ‘Rejina’, although the Pn of ‘Rejina’ was higher than that of ‘Micro-Tom’ (**Figure 1**). This discrepancy may be attributed to two reasons. Firstly, the respiration rate of ‘Rejina’ was higher than that of ‘Micro-Tom’ (**Supplementary Figure 6**), leading to more dry mass being consumed during the dark period. Secondly, there were about ten true leaves in ‘Rejina’ and six true leaves in ‘Micro-Tom’ on the main stem. The fifth true leaf from the bottom in ‘Rejina’ and the sixth true leaf in ‘Micro-Tom’ were expanded at 36 DAS (**Supplementary Figure 7A**). The age of the top leaf in ‘Rejina’ was younger than that in ‘Micro-Tom’ at the same DAS. In addition, until harvest, the age of the bottom leaf in ‘Micro-Tom’ was younger than that in ‘Rejina’. Furthermore, the top leaves (2–3 leaves) in ‘Rejina’ occupied 20–30% and in ‘Micro-Tom’ occupied 30–50%. Therefore, the Pn of the whole canopy in ‘Rejina’ may be less than that in ‘Micro-Tom’. In the future, the Pn of the whole canopy should be measured and used for the investigation of the RUE of the canopy. Moreover, the growth speed was also different between the two cultivars. Until 36 DAS, when the first flower in ‘Micro-Tom’ bloomed, the ‘Micro-Tom’ plant was taller and larger than the ‘Rejina’ plant (**Supplementary Figure 7A**). However, the ‘Rejina’ plant was taller and larger than the ‘Micro-Tom’ plant at 50 DAS (**Supplementary Figure 7B**). Therefore, it is also important to consider choosing tomato cultivars with high RUE in the commercial PFAL.

### 4.3 White light may have a greater capacity for enhancing FBRUE than red and blue light in a PFAL

LED fixtures for horticulture generally comprise a combination of LEDs emitting red (approx. 660 nm), blue (approx. 450 nm), white, and/or far-red (approx. 730 nm) light because of their high efficiency and efficacy (Kusuma et al., 2020). Theoretically, the photon efficacy (µmol J^−1^) of blue LEDs is less than that of red LEDs (Tsao et al., 2010). Additionally, the photosynthetic efficiency of blue photons is at most 20% lower than that of photons from a typical red LED (660 nm) (McCree, 1971). Therefore, more red light usually leads to higher energy use efficiency. However, a blue light percentage of 5–22 or 30% is typically employed to prevent excessive stem elongation and shade-avoidance characteristics (Hogewoning et al., 2010; Kusuma et al., 2020). Therefore, in the present study, the proportion of blue light we selected did not exceed 25% (**Supplementary Table 2**). Moreover, a luminescent material coating that absorbs blue photons and luminesces at longer wavelengths is used to construct white LEDs. Hence, the photon efficacy of white LEDs is less than that of blue and red LEDs. However, the advantages of white LEDs, such as affordability, wide spectrum range, and enhanced comfort in the workplace, have made them increasingly popular in PFALs.

In the present study, white light increased FBRUE (**Figure 5**) by increasing RUE (**Figure 3**) and F_fruits_ (**Figure 4**). There may be two possible reasons why RUE in WH was the highest. First, the changing trend of RUE under different light qualities was associated with Pn changes in the two cultivars. The Pn in WH was high until 67 DAS in both cultivars (**Figure 1**). In this study, the white LED light had 3.3% far-red photons (**Supplementary Table 2**) that may increase Pn. Zhen and van Iersel (2017) and Murakami et al. (2018) reported that supplementing far-red photons (peaking at 735 nm) to existing red+blue or white LED light synergistically enhanced the quantum yield of PSII and the Pn of leaves in a broad range of light intensities. Second, the white light in WH in this study had 46.7 % green light, while other light treatments had less than 1% green light (**Supplementary Table 2**). Green light is able to penetrate into the leaves more deeply than both red and blue lights, thus enabling leaves in the lower canopy to absorb more of the green light (Terashima et al., 2009). Additionally, the efficiency of photosynthesis is known to be highly driven by the absorption of green light in leaves (Björkman, 1968; McCree, 1972b). More green light in WH might enhance the canopy RUE by improving the uniformity of light distribution throughout the canopy.

The F_fruits_ in WH was significantly higher than those in other treatments at 100 DAS in ‘Rejina’ (**Figure 4B**). A higher red/blue light ratio led to higher dry mass partitioning to leaves in tomatoes under red and blue LED light (Liang et al., 2021). The shoot-root ratio in tomatoes (cv. Sida) was higher under high red-to-blue ratio light, which was in agreement with Thwe et al. (2020). In the present study, the dry mass partitioning to leaves in both R and R9B1 was higher than that in WH and R3B1 in ‘Micro-Tom’ at 64 DAS (data not shown). In addition, far-red light promotes fruit growth by increasing dry mass partitioning to fruits (Ji et al., 2019). This may be one reason why F_fruits_ was the highest at 90 and 100 DAS in ‘Rejina’ (**Figure 4B**).

In ‘Micro-Tom’, the lowest Pn in R (**Figure 1A**) led to the lowest fruit dry weight, which resulted in the lowest fruit dry matter ratio (**Table 4**). However, red light may improve the content of soluble sugars in tomatoes (Erdberga et al., 2020). This aligns with a previous study indicating that an increased red-to-blue ratio enhanced tomato glucose and fructose contents and sugar/acid ratio (Thwe et al., 2020). Therefore, the fruit Brix in R3B1 rather than R was the lowest (**Table 4**). In addition, the fruit dry matter ratio and Brix in WH were the highest among all treatments at 82 DAS. This may be attributed to white light containing far-red radiation that can increase fruit sugar concentration (Ji et al., 2020). The expression of genes related to both sugar transportation and metabolism, such as *HY5* (van Gelderen et al., 2018), *SWEET11*, and *SWEET12* (Chen et al., 2016), were increased by far-red light. In summary, white light is suitable for enhancing RUE, FBRUE, and fruit quality.

This study has certain limitations. It is important to note that white light is a mixture of light. In this study, we used just one kind of white light. Therefore, other kinds of white light with different spectra emitted by white LEDs with different color temperatures should be investigated in the future to determine whether white light has a greater capacity for enhancing the FBRUE of tomatoes than red and blue light in a PFAL. In addition, the cool-type white LED with a lower R/B light ratio may improve RUE and FBRUE of dwarf tomatoes at the reproductive growth stage. In *Arabidopsis*, a blue light (470 nm) threshold intensity of 5 μmol m^–2^ s^–1^ was found to activate *psbD*, a PSII core protein D2-encoding gene through cryptochromes (Mochizuki et al., 2004). In addition, there may be a qualitative or threshold effect of blue photons on leaf photosynthesis in cucumbers (Hogewoning et al., 2010). However, the relationship between blue light and RUE/FBRUE is still unclear, in terms of whether there is a qualitative threshold and/or quantitative progressive effect.

## 5 Conclusions

Our study showed that the decrease in RUE was ultimately caused by the monochromatic red light, which increased SLA, reflectance, and transmittance but decreased absorptance and Pn. Additionally, a higher blue light proportion, up to 25%, led to higher Pn, which further caused higher RUE under the combined red and blue light. Moreover, blue light improved FBRUE by enhancing RUE rather than F_fruits_. We also found that FBRUE is cultivar-specific and was higher in ‘Micro-Tom’ than in ‘Rejina’. This distinction was attributed to RUE rather than F_fruits_.

Compared with red and blue light, white light increased FBRUE by 0.14–0.25 g mol^−1^. In both cultivars, white light improved RUE. However, F_fruits_ was increased by the white light only in ‘Rejina’. Moreover, white light improved fruit dry matter ratio and Brix in ‘Micro-Tom’, and this effect was also cultivar-specific. In summary, white light has more potential to enhance FBRUE than red and blue light by improving RUE and F_fruits_; hence, it is recommended for improving RUE, FBRUE, and fruit quality at the reproductive growth stage. Our study results will be helpful in comprehending how light quality affects the RUE and FBRUE of dwarf tomatoes in PFALs. Further studies are essential to determine what kinds of white light have more potential to boost the FBRUE of tomatoes than blue and red light, and whether blue light intensity has a qualitative threshold effect on FBRUE in tomatoes.

## Data availability statement

The original contributions presented in the study are included in the article/**Supplementary Material.** Further inquiries can be directed to the corresponding author.

## Author contributions

EG: Conceptualization, Funding acquisition, Methodology, Supervision, Writing – review & editing. XK: Conceptualization, Formal analysis, Investigation, Methodology, Validation, Visualization, Writing – original draft. HY: Writing – review & editing. SH: Writing – review & editing.
