# Supplementary material for: Effect of red and blue light versus white light on fruit biomass radiation-use efficiency in dwarf tomatoes
Source: Front Plant Sci. 2024 Jun 21;15:1393918. doi: 10.3389/fpls.2024.1393918 (PMC11224545; doi:10.3389/fpls.2024.1393918)
Supplement: Supplementary file 1 [file DataSheet_1.docx]

Supplementary Material

**Effect of Red and Blue Light versus White Light on Fruit Biomass Radiation-Use Efficiency in Dwarf Tomatoes**

**Xinglin Ke, Hideo Yoshida, Shoko Hikosaka and Eiji Goto^*^**

*** Correspondence:** Eiji Goto: goto@faculty.chiba-u.jp

# Supplementary Figures and Tables

## Supplementary Figures

##
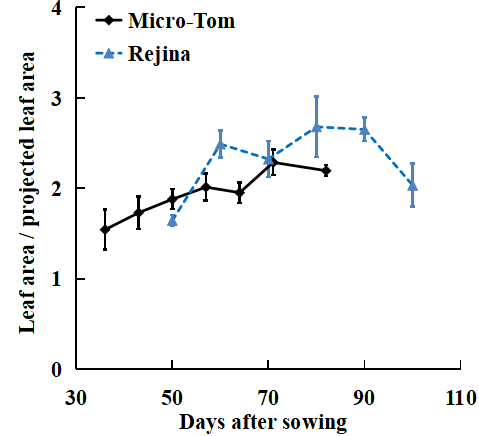


## Supplementary Figure 1. Leaf area / projected leaf area changes of two dwarf tomato cultivars over time. Each value represents the average of 12 values (n = 12). Error bars represent ± standard error.

**
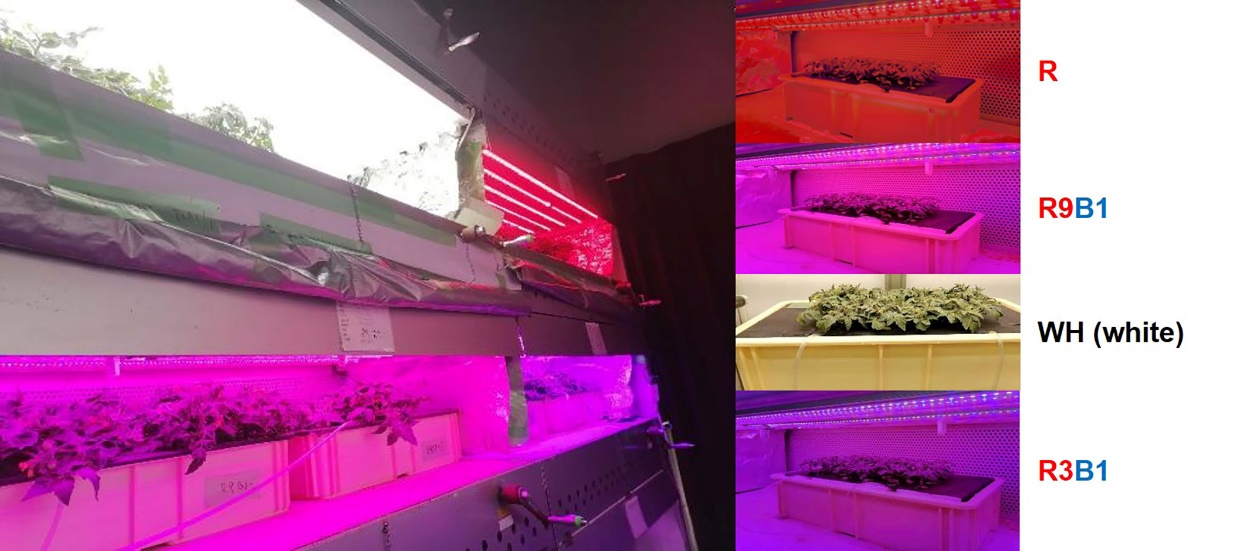
**

**Supplementary Figure 2.** **Four light treatments in a cultivation room.** Red light (R), white light (WH), and the mixture of red and blue lights: red/blue light ratio = 3 (R3B1) and red/blue light ratio = 9 (R9B1).

**Supplementary Figure 3.** **Spectral photon flux distributions of red LED lamp (A), red and blue LED lamps in R9B1 (B), white LED lamp (C), as well as red and blue LED lamps in R3B1 (D).** R3B1 and R9B1 represent photon flux ratios (red to blue light) of 3:1 and 9:1, respectively. The peak wavelengths of the white lamp were 446 nm and 592 nm. The peak wavelengths of red and blue light were 667 and 450 nm, respectively. The maximum value of photon flux was converted to 1.0.

**Supplementary Figure 4. Relationships between accumulated dry weights (ΔW) and cumulative intercepted PPFDs (ΔI_PPFD_) per plant in ‘Micro-Tom’ under different light qualities.** (A), (B), and (C) were the results of three repetitions, respectively. The slope of the fitted linear relationship is the RUE at the reproductive growth stage. R: red light; R9B1: red/blue light ratio = 9; WH: white light; R3B1: red/blue light ratio = 3.

**Supplementary Figure 5. Relationships between accumulated dry weights (ΔW) and cumulative intercepted PPFDs (ΔI_PPFD_) per plant in ‘Rejina’ under different light qualities.** (A), (B), and (C) are the results of three repetitions, respectively. The slope of the fitted linear relationship is the RUE at the reproductive growth stage. R: red light; R9B1: red/blue light ratio = 9; WH: white light; R3B1: red/blue light ratio = 3.

**
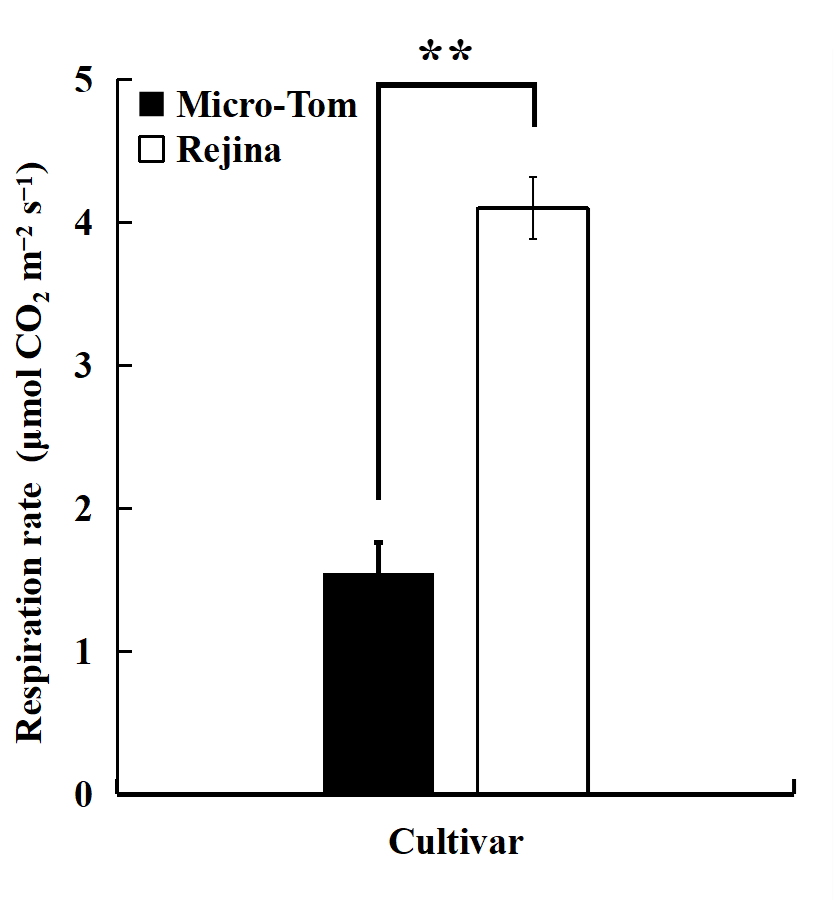
**

**Supplementary Figure 6. Respiration rates of leaves in two tomato cultivars.** The respiration rates of top true leaves were measured at 50 DAS in WH treatments in both cultivars. ** indicates a significant difference among the treatments based on the Student’s test at *p* < 0.01 (n = 4).

**
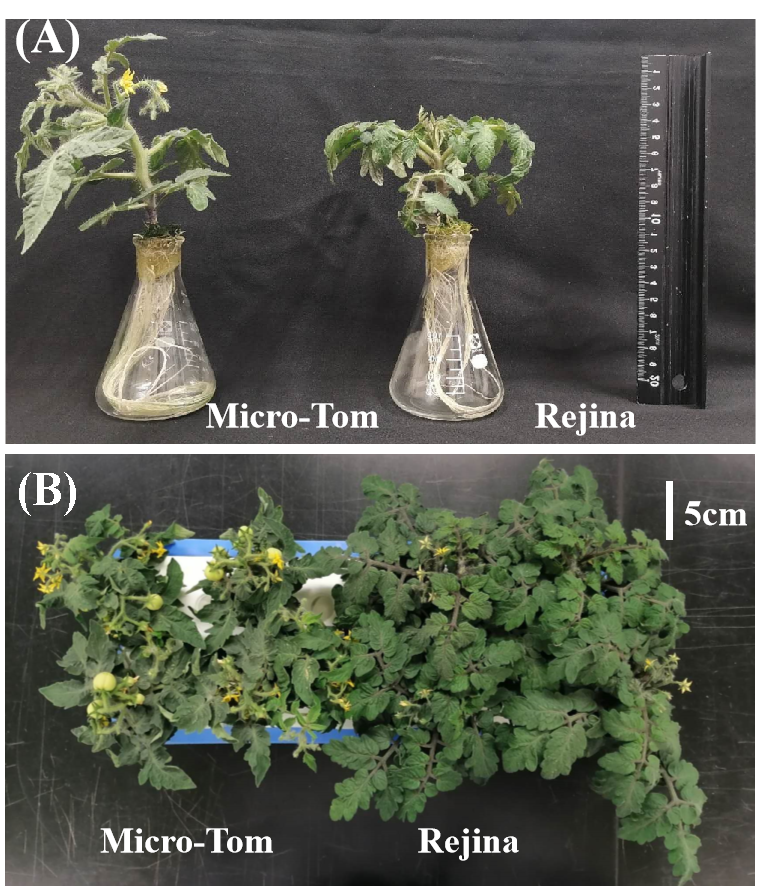
**

**Supplementary Figure 7. ‘Micro-Tom’ and ‘Rejina’ plants at 36 DAS (A) and 52 DAS (B) under white LED light.**

## Supplementary Tables

**Supplementary Table 1. The number of days, plant density, used lamps, and photosynthetic photon flux density (PPFD) on the top of the canopy during different growth periods in two cultivars.**

| **Cultivar** | **Growth period**  **(DAS)** | **The number of days** | **Plant density (plant/m^2^)** | **PPFD**  **(µmol m^−2^s^−1^)** | **Lamp** |
| --- | --- | --- | --- | --- | --- |
| Micro-Tom | 0–23 | 23 | 800 | 200 | White LED lamps^1^ |
|  | 24–35 | 11 | 476.2 | 300 | Red and Blue LED lamps^2^ |
|  | 36–82 | 47 | 238.1 | 300 | Depend on the treatment |
| Rejina | 0–23 | 23 | 800 | 200 | White LED lamps^1^ |
|  | 24–43 | 19 | 261 | 300 | Red and Blue LED lamps^2^ |
|  | 44–49 | 5 | 55.9 | 300 | Red and Blue LED lamps^2^ |
|  | 50–100 | 50 | 37.3 | 300 | Depend on the treatment |

^1^ White LED light lamps (LDL40S-N19/21, Panasonic Corporation., Osaka, Japan).

^2^ Red and blue LED lamps (CIVILIGHT, DPT2RB120Q33 40 type, Showa Denko K.K., Tokyo, Japan; R:B = 9:1).

**Supplementary Table 2.** **Spectral data for LED lamps at the wavelength of 400–700 nm.** ‘%’ represents the ratios of blue, green, red, and far-red photon fluxes as a percentage of photon flux density. The R/B ratio represents the photon flux ratio of red light to blue light. ‘R’ respects red LED lamps. ‘WH’ represents white LED lamps. R3B1 and R9B1 represent red-blue LED lamps with photon flux ratios of 3:1 and 9:1, respectively, of red-to-blue light. The phytochrome photostationary state (PSS) is calculated using the spectral composition and intensity of light received by plants.

|  | **R** | **R9B1** | **WH** | **R3B1** |
| --- | --- | --- | --- | --- |
| % Blue (400–499 nm) | 0.2 | 9.9 | 17.1 | 24.5 |
| % Green (500–599 nm) | 0.5 | 0.4 | 46.7 | 0.4 |
| % Red (600–699 nm) | 98.3 | 89.0 | 32.9 | 74.7 |
| % Far-red (700–800 nm) | 1.0 | 0.7 | 3.3 | 0.4 |
| R/B ratio | - | 9.0 | 1.9 | 3.0 |
| PSS | 0.88 | 0.88 | 0.85 | 0.87 |

**Supplementary Table 3. Effects of light quality on fruit fresh, dry weights, and yield in ‘Micro-Tom’ 71 DAS and in ‘Rejina’ 70 DAS.** Each value represents the mean ± standard error. Different letters indicate significant differences at the *p* < 0.05 level among light-quality treatments with Tukey−Kramer’s test (n = 3−4). R: red light; R9B1: red/blue light ratio = 9; WH: white light; R3B1: red/blue light ratio = 3.

| **Cultivar** | **Treatment** | **Fruit fresh weight**  **(g)** | **Fruit dry weight**  **(g)** | **Yield**  **(g FW m^−2^)** |
| --- | --- | --- | --- | --- |
| Micro-Tom | R | 49.4 ± 8.0 | 4.0 ± 0.8 | 11.8 ± 1.9 |
|  | R9B1 | 55.5 ± 6.0 | 4.8 ± 0.3 | 13.2 ± 1.4 |
|  | WH | 50.2 ± 9.2 | 4.3 ± 0.8 | 11.9 ± 2.2 |
|  | R3B1 | 69.6 ± 11.8 | 6.1 ± 0.9 | 16.6 ± 2.8 |
| Rejina | R | 33.0 ± 2.0 c | 4.7 ± 1.1 b | 2.7 ± 0.6 b |
|  | R9B1 | 73.0 ± 17.4 b | 4.7 ± 0.6 b | 2.5 ± 0.3 ab |
|  | WH | 135.7 ± 21.3 a | 9.2 ± 1.5 a | 5.1 ± 0.8 a |
|  | R3B1 | 90.9 ± 7.6 b | 6.0 ± 0.4 ab | 3.4 ± 0.3 ab |

**Supplementary Table 4. Effects of light quality on the number of fruits, fruit fresh, and dry weights in ‘Micro-Tom’ 82 DAS and in ‘Rejina’ 100 DAS.** Each value represents the mean ± standard error. Different letters indicate significant differences at the *p* < 0.05 level among light-quality treatments using Tukey−Kramer’s test (n = 3−4). R: red light; R9B1: red/blue light ratio = 9; WH: white light; R3B1: red/blue light ratio = 3.

| **Cultivar** | **Treatment** | **Number of fruits** | **Fruit fresh weight**  **(g)** | **Fruit dry weight**  **(g)** |
| --- | --- | --- | --- | --- |
| Micro-Tom | R | 15.0 ± 1.2 | 79.1 ± 8.1 | 6.2 ± 0.9 |
|  | R9B1 | 12.8 ± 1.3 | 72.6 ± 7.8 | 5.9 ± 0.5 |
|  | WH | 14.3 ± 1.9 | 66.8 ± 10.4 | 6.4 ± 1.0 |
|  | R3B1 | 15.8 ± 1.5 | 90.5 ± 4.7 | 7.7 ± 0.4 |
| Rejina | R | 36.3 ± 2.2 | 212.3 ± 73.4 | 13.3 ± 4.2 |
|  | R9B1 | 34.7 ± 5.4 | 245.9 ± 77.4 | 16.2 ± 5.2 |
|  | WH | 31.0 ± 5.5 | 339.5 ± 42.3 | 23.3 ± 3.2 |
|  | R3B1 | 30.0 ± 5.6 | 300.2 ± 87.0 | 20.3 ± 5.6 |
